# An Account of a Fifteen Years Pregnancy, Where the Child Was Contained in the Urinary Bladder

**Published:** 1805-12

**Authors:** 

**Affiliations:** Rostock


### *An Account of a Fifteen Years Pregnancy, where the Child was contained in the Urinary Bladder; by* Professor Josephi, *of Rostock.*

( Communicated by Dr. Arneman. )

A Woman, 47 years of age, tall and well made, and in good health from her infancy, after having been once well delivered, became pregnant the second time. A few weeks after conception she had her menses, and this continued, a few intervals excepted, till the seventeenth week of her pregnancy. During the first period she complained much of sickness, vomiting, and sometimes obstruction of the body. At the usual time she felt the movement of the child very plainly. After the first half of the pregnancy was passed, she became affected with violent colic, and most excruciating pains, during which she was thrown upon a bed by a woman. Since this time she constantly felt, on the right side, at the annulus abdominalis, a very disagreeable sensation whenever the child moved, as well as during the period of its growth. Sometimes she experienced spastic constrictions almost to her toes. In the course of the thirty-seventh week, towards evening, she complained complained of violent pains, with shiverings, and a sensation as if the bowels were drawn downwards. The abdomen was much swelled, and the pains rising to a most excruciating height, ended at last in a palsy of the right leg, which continued three weeks. After this time she no longer felt the movement of the child, nor has she ever been well. Her breasts were filled with milk.

Towards the fifteenth month, a discharge of corrupted or putrid blood was observed from the genitals. A month after, her menses again appeared, and lasted from this time during her life, yet not quite regularly. The general state of her health occasionally improved, and excepting frequent pains in her bowels, and a pressure on the urinary bladder, she felt herself tolerably well. In this state she remained nine years, when the direful catastrophe began. After having experienced several violent frights, she had a severe fit of shivering and colic, which affected the whole body, and terminated in pains like those of women in labour; these continued several hours; and the next morning the patient found herself affected with the ague, which lasted a quarter of a year. From this period she suffered from a painful ischuria, with violent spasms in the abdomen, and seldom experienced even a few minutes easiness. Being constantly under the necessity of making water, she was obliged to rest on her knees, or to be in a forward bent posture; the quantity of urine was small, resembling a whitish purulent matter, and often mixed with pieces of a thick jelly.

After three years had elapsed, she observed a hard body in her urethra; and at the expiration of a few months she evacuated with the urine a greyish stone, of the size of a horse bean, which gave her some relief. Consulting with her physician, he administered various medicines; in consequence of which, a bone, by appearance the tibia of a child, passed off with the urine, under violent agonies. A few days after another stone came off, and a piece of the bones of the head. In this manner she brought away by degrees ninety-four stones of different size, the largest resembling a bean; they were differently formed, but all of a white colour; likewise a number of bones of the head, some vertebra, six teeth, part of the fibula, and twenty-one flat pieces of bone.

In about a year after, a bone stopped the mouth of the urethra, and caused almost insupportable pain; her physician therefore resolved to make an incision in the urethra, after which he extracted the lower jaw bone, and the the patient obtained some relief. But all her sufferings increasing from time to time, she went to Rostock, and Professor Josephi was consulted. All the physicians residing at this place were called in, and they agreed that the child was contained in the urinary bladder, and nothing but an operation could save the patient. It was, therefore, resolved that by making an incision in the urethra and the neck of the bladder, the patient might be delivered.

By an exact examination, the abdomen was not at all distended, only close above the bones of the pubis, and more toward the right side, the hard parts of the child were very plainly felt; and by pressing this part the patient always experienced very sensible pains. The genitals were well formed, only excoriated by urine. The pelvis was well shaped, the vagina more contracted, and the uterus small, empty, of its natural form, and drawn up very high. The bladder was much extended, and the hard parts it contained could be plainly felt. The urethra was likewise extended, and at the orifice swelled and tumid. The colour of the patient was pale and cachectic, and her sufferings beyond description. Always laying on her knees, she never enjoyed more than a few minutes rest or sleep; her appetite was pretty good.

In order to afford the patient some relief, the external application of the volatile ointment with opium, and lenient warm cataplasms, were ordered. Inwardly, the extract of henbane and the laudanum liquidum Sydenhami. With the assistance of a catheter the urine was drawn off with difficulty: also lenient glysters were applied. Every trial to take away the different parts contained in the bladder with instruments was fruitless. The instrument always slipped off, and it was impossible to catch any of the parts; besides, the application of the instrument always increased the spastic affections. No other resource was left, but to make an incision, and to try by this mode to obtain the extraction of the parts.

It was thought advisable to make the incision in the urethra and the neck of the bladder, but Prof. Josephi preferred the incision above the os pubis. The operation was performed in the presence of five physicians and several surgeons.

The patient was previously ordered to take an opening glyster, and to drink freely of barley water. During the operation she was placed horizontally upon beds on a table, the head a little elevated, the knees rather bent and distended, the operator standing between them. A closed catheter catheter was applied in the bladder. The incision was made above the arcus ossis pubis, towards and through the linea alba, about three inches long, one inch below the adhesion of the peritonæum; the knife was thrown in the bladder, so that the point of the instrument and the finger of the left hand entered together. The bladder was not as in the natural state, membranous and thin, but thick, hard, and fleshy. At the opening the urine passed out; the catheter was opened directly, and the incision in the bladder dilated about an inch long. The Doctor found the whole bladder entirely filled with hard and pointed pieces of bone, besides a number of soft parts. The incision therefore was about an inch more dilated, and one hundred and twelve pieces of bone, partly entire, and partly corroded, were taken out; besides, twenty pieces of stony concrements, some cartilages, part of the skull and face, and the bowels, of a dark blue colour, adhering to the omentum and mesenterium. The femoral bones were about three inches and a quarter long, other bones were covered with a calcareous concrement, and several of them adhered so firmly to the inner coat of the bladder, that they could not be taken away but by force. The inner surface of the bladder was rough, thick, uneven, spongy, and totally different from its natural texture. At the bottom of the bladder an opening was to be felt, and in putting the fingers through it touched the bowels of the patient.

During the operation the unfortunate woman remained very quiet, notwithstanding violent spasms in the abdomen commenced, during which the bladder was contracted, like the uterus in labour. The whole operation was performed in forty-two minutes, and scarcely four ounces of blood were lost. The dressing consisted of ung. simplex spread upon lint, and a soft compression fastened with the T bandage; the wound of the integuments was contracted with strips of adhesive plaster.

After the operation the patient did pretty well; she complained of no particular pain, and desired to look at the different contents taken from her. She took some water-soup, and gruel was ordered for her drink. In the afternoon she complained of cold, and a painful sensation in the wound, more towards the right side; her pulse was from 85 to 95, quick, and spastic, and the whole body covered with a cold perspiration. A sedative ointment was applied to the body, and a saline draught with the addition of opium prescribed. The pain was diminished, as as well as the spasms, and she enjoyed some sleep at intervals. Through the catheter some bloody urine went off. During the night she slept about an hour, and, on awaking, complained of colic and hiccup.

The next morning her pulse was 90, and very thin; the hiccup sometimes was so violent that she was thrown upwards. The body was uniformly warm, the abdomen not painful nor hard. She perspired a little; the urine went through the catheter, but the body was constipated, and different glysters were applied without any effect. At noon she complained of a pressure at the scrobiculum cordis; the pulse became very quick and small, and she vomited a good deal of slime. In the evening she became very weak, was always slumbering, and sometimes convulsed. An emulsion with camphor was prescribed, and glysters of valerian, &c.

On the third day, at five o'clock in the morning, the body became loose with most fœtid dejections. Her face was entirely altered, and every symptom of approaching death appearing; she expired the same morning at eight o'clock.

The next day the section was made. The abdomen was much distended and hard; the wound shewed a good appearance, and likewise the genitals; the omentum and mesenterium were without fat, and empty of blood, but no sign of inflammation or gangrene. The liver was of an ash colour with several spots. The vesicula fellis much extended with gall and stony concrements. There was no blood extravasated, nor any urine in the cavum abdominis, but about four ounces of a yellowish, thin, and bad smelling matter was contained in the pelvis, towards the right side. The wound in the bladder was about two inches, and much distended. The whole bladder had a thin, spongy, and in many parts cartilaginous texture, with a great many spongy excrescences; at the upper part a hole was found, about the size of a six-pence, hard and callous. Towards the right side a bag of three inches was observed, adhering to the bladder, and containing part of the bowels (intestina tenuia). By examining these parts minutely, another opening at the other side of the bladder was detected, about two inches and a half in diameter. Thro' this hole part of the bowels passed into the bladder; it connected with the peritoneum, which formed a separate bag in the bladder, the surface of which was smooth and covered with a purulent matter. The uterus was quite natural, also the tuba and the ovarium on the left side; at the the right the tube was wanting, only a remaining part adhered firmly to the bladder. The right ovarium was likewise deficient, only the vessels of this part remaining. All the other parts of the abdomen were sound, and in their natural state. Prof. Josephi draws this conclusion, that, at the beginning, the pregnancy had taken place in the right ovarium; that the pieces of jelly, which the patient evacuated with the urine, were the dissolved soft parts of the fœtus; and that the stony concrements were formed from the dissolved bones, according to chemical analysis, and consisted entirely of phosphat of lime.

